# Application of a new dietary pattern analysis method in nutritional epidemiology

**DOI:** 10.1186/s12874-018-0585-8

**Published:** 2018-10-29

**Authors:** Fengqing Zhang, Tinashe M. Tapera, Jiangtao Gou

**Affiliations:** 1 Department of Psychology, Drexel University, Philadelphia, PA 19104 USA; 2 Department of Biostatistics and Bioinformatics, Fox Chase Cancer Center, Temple University Health System, Philadelphia, PA 19111 USA

**Keywords:** Dietary pattern analysis, LASSO, Principal component analysis, Cardiovascular disease, Food-frequency questionnaire, NHANES

## Abstract

**Background:**

Diet plays an important role in chronic disease, and the use of dietary pattern analysis has grown rapidly as a way of deconstructing the complexity of nutritional intake and its relation to health. Pattern analysis methods, such as principal component analysis (PCA), have been used to investigate various dimensions of diet. Existing analytic methods, however, do not fully utilize the predictive potential of dietary assessment data. In particular, these methods are often suboptimal at predicting clinically important variables.

**Methods:**

We propose a new dietary pattern analysis method using the advanced LASSO (Least Absolute Shrinkage and Selection Operator) model to improve the prediction of disease-related risk factors. Despite the potential advantages of LASSO, this is the first time that the model has been adapted for dietary pattern analysis. Hence, the systematic evaluation of the LASSO model as applied to dietary data and health outcomes is highly innovative and novel. Using Food Frequency Questionnaire data from NHANES 2005–2006, we apply PCA and LASSO to identify dietary patterns related to cardiovascular disease risk factors in healthy US adults (*n* = 2609) after controlling for confounding variables (e.g., age and BMI). Both analyses account for the sampling weights. Model performance in terms of prediction accuracy is evaluated using an independent test set.

**Results:**

PCA yields 10 principal components (PCs) that together account for 65% of the variation in the data set and represent distinct dietary patterns. These PCs are then used as predictors in a regression model to predict cardiovascular disease risk factors. We find that LASSO better predicts levels of triglycerides, LDL cholesterol, HDL cholesterol, and total cholesterol (adjusted *R*^2^ = 0.861, 0.899, 0.890, and 0.935 respectively) than does the traditional, linear-regression-based, dietary pattern analysis method (adjusted *R*^*2*^ = 0.163, 0.005, 0.235, and 0.024 respectively) when the latter is applied to components derived from PCA.

**Conclusions:**

The proposed method is shown to be an appropriate and promising statistical means of deriving dietary patterns predictive of cardiovascular disease risk. Future studies, involving different diseases and risk factors, will be necessary before LASSO’s broader usefulness in nutritional epidemiology can be established.

## Background

With the global prevalence of chronic diseases increasing, it is now widely accepted that diet has an important role to play, as many of these diseases are affected by unhealthy dietary habits [1, 2]. A set of studies across cohorts [3–5] found that higher diet quality (top quintile) was consistently associated with an 11–28% reduced risk of death from all causes, cardiovascular disease, and cancer when compared with the lowest quintile. This association was observed independent of known confounders. Given these disparities, it is essential to examine dietary intake in greater detail. Doing so will help to establish the role of diet in chronic diseases, assist in the development of targeted disease prevention initiatives, and improve the effectiveness of public health recommendations [6].

The complexities of nutrient intake and metabolism make the relationship between diet and health a multifaceted one. To capture the multidimensionality of diet, studies of the overall diet examine the combined effect of total dietary intake. This is important, since dietary components are consumed in combination and correlated with one another [7]. Complex and cumulative effects cannot be sufficiently captured by examining dietary components in isolation.

Currently, methods utilized by researchers in this field fall into two categories: *investigator-driven* and *data-driven* [7]. The investigator-driven approach defines scores or indices of dietary quality based on a priori guidelines in the literature, then uses these measures to predict health status. The Healthy Eating Index (HEI), for example, has been used in the literature to summarize dietary quality [8], and has been shown to correlate with nutritional biomarkers [9]. In the data-driven approach, dietary patterns are empirically derived from the underlying dietary data using statistical models, such as principal component analysis (PCA), factor analysis, cluster analysis, and *k*-means, among others [1, 10].

The implementation of the investigator-driven approach is simple and reliable. In some cases, however, the indices defined by the literature are unable to predict the current prevalence of diet-related chronic disease with sufficient accuracy [7]. This problem arises partly because dietary quality scores combine many individual foods without considering the correlated nature of the components. For example, people with a midrange HEI score can have very different contributing dietary constituents – whether the individual constituents are all in the midrange, or whether some components are high while others are low. Additionally, dietary scores constructed by health professionals may miss important patterns of nutritional intake that affect cardiometabolic risk factors (e.g., blood lipids or glycemic control) and could be identified by a data-driven approach.

Meanwhile, data-driven methods have become popular among machine learning professionals, who have deployed such methods with increasing success. Cluster analysis and *k*-means, for example, can be used to empirically discover subgroups of participants with distinct dietary patterns [11–15], while factor and principal component analyses can be used to reduce the number of variables (e.g., individual foods) in a data set to those which explain most of the variability in the data [11, 16]. These methods, however, have been found to be sensitive to extrema and to have relatively low prediction accuracy [17, 18]. Additionally, these methods are not entirely data-driven: rather, they require multiple decisions from the investigators (e.g., the number of groups in *k*-means and the choice of the metrics quantifying group dissimilarity). These decisions may vary by investigator and hence the empirically derived dietary patterns are not entirely data driven, which could lead to problems in the validity and reproducibility of these pattern analysis models [7, 12].

In order to enhance the prediction of clinically meaningful risk factors and the identification of predictive dietary patterns, we propose a new dietary pattern analysis method using the advanced LASSO (Least Absolute Shrinkage and Selection Operator) model [19]. LASSO is a regression-based method that penalizes the absolute value of the regression coefficients; in doing so, it regularizes the impact a coefficient may have in the overall regression [19]. The greater the penalization, the greater the shrinkage of a coefficient, with some coefficients shrinking to zero. The LASSO model is hence a form of automatic feature selection, an approach which has found successful application in such fields as neuroimaging, genomics, and computational chemistry [20–22]. Despite the potential advantages of LASSO, this is the first time the model will be applied to the identification and examination of dietary patterns predictive of health outcomes. Hence, the systematic evaluation of the LASSO model in analyzing dietary data related to health outcomes is considered highly innovative and novel.

The following paper describes an attempt to predict risk factors for cardiovascular disease using the LASSO model on food intake data collected through the Food Frequency Questionnaire. In this instance, risk is quantified as the levels of triglycerides, LDL cholesterol, HDL cholesterol, and total cholesterol in the blood. In addition, we compare the predictive performance of LASSO against the traditional dietary pattern analysis method utilizing linear regression, applying the latter to components derived from PCA.

## Methods

### Data set

Subjects in this study were participants in the National Health and Nutrition Examination Survey (NHANES) from 2005 to 2006. As part of the nationwide survey, which involves about 5000 persons each year, participants completed the Household Adult Questionnaire and the Dietary Food Frequency Questionnaire. Additionally, participants were invited to a mobile examination center, where blood and urine specimens were obtained, and a number of tests and measurements—including body measurements and blood pressure testing—were performed [23].

### Analytic sample

A number of restrictive criteria were applied to the data. Participants in our final data set were at least 20 years old; were not pregnant or lactating at the time of data collection; were not currently suffering from heart disease; and were not diabetic. Moreover, our data set included only participants who had completed the Food Frequency Questionnaire. The resulting sample included 2609 participants with a mean age of 49.56 and a mean BMI of 28.45 kg/m^2^. 54% of the participants in our sample were female. Laboratory measures of biomarkers for cardiovascular disease (triglycerides, LDL cholesterol, HDL cholesterol, and total cholesterol) were obtained from NHANES.

### Dietary assessment methods

Participants in the NHANES study completed a full Food Frequency Questionnaire (FFQ), which collects quantitative information about how many times foods had been consumed in the past month. FFQ has been validated to be a reasonable tool to assess relationships between dietary habits and cardiovascular risk factors [6, 24]. Following the recommendation in the literature [6], the individual food items and beverages measured in FFQ were combined into 35 food groups that represented all major food groups consumed by the US population. FFQ raw scores were available from NHANES website.

### Laboratory methods

The NHANES dataset also includes significant laboratory test results. Among these were blood tests, which were conducted via venipuncture, with necessary precautions taken to ensure participants could safely complete the test. Blood levels of triglycerides, LDL cholesterol, HDL cholesterol, and total cholesterol were available from NHANES.

### Statistical analysis

#### Statistical software

Data preparation and analysis were completed in R Statistical Software (version 3.2.3 [2015-12-10]). Because NHANES studies tend to oversample underrepresented ethnicities, NHANES provides guidelines for sample weighting and stratification in the data set. LASSO is available in the “glmnet” R package (Version 2.5). Appropriate sampling weights were used in all statistical analyses to account for the unequal probability of selection, noncoverage, and nonresponse bias. The “survey” package in R allows for principal component analysis, automatically incorporating the sampling weights provided by NHANES. Before we applied the LASSO model, each observation was weighted by its sampling weight as recommended by NHANES [23].

#### Statistical methods

Principal Component Analysis (PCA) is a popular analytical tool used commonly in dimension-reduction problems. Consider a data matrix X wherein each of the *n* rows represents an observation and each of the *p* columns represents a variable or feature. The transformation that PCA utilizes is *p*-dimensional vectors of loadings that map old data points to new data points along principal components. The first principal component explains the largest possible variance; each succeeding component accounts for the highest possible remaining variance, under the constraint that it is orthogonal to preceding components.

The LASSO model is a shrinkage method, inspired by stepwise selection, that actively selects for a subset of predictors for regression; this results in a more interpretable and relevant set of predictors [19]. LASSO performs similarly to the ridge regression approach, which shrinks regression coefficients in order to reduce the likelihood of overfitting. In the case of LASSO, however, this shrinkage technique is computed so as to shrink the sum of the absolute value of regression coefficients, forcing some of the coefficients to zero and hence selecting for non-zero features. Ridge regression, by contrast, is unable to shrink coefficients to exact zeros. In this sense, LASSO is a sparse model.

Dietary data and outcome data were inspected visually and found to be positively skewed. Consequently, the data were truncated at 4 standard deviations above the mean and log-transformed, as suggested by Kerver et al. [6]. We applied principal component analysis with varimax rotation on the frequency of consumption of the defined food groups. The number of components was selected based on the scree plot, eigenvalues, and parallel analysis, as well as a consideration of content and theoretical consistency. The selected principal components were then used as predictors in multiple linear regression models. Four regression models were built separately to predict levels of triglycerides, LDL cholesterol, HDL cholesterol, and total cholesterol. In addition, we applied LASSO to the transformed food-group data to predict each of the four biomarkers for cardiovascular disease. Data were randomly split into a training set (approximately 70% of the data) and an independent test set. The training set was used to build the model and the test set was used to evaluate the model performance. Model evaluation using an independent test set was an objective way to examine how well the trained model could be generalized to a new set of data. Correlation coefficients and adjusted *R*^2^ for each model on the test data were computed for model comparison. Covariates such as age, sex, BMI, income, and ethnicity were controlled for in all analyses.

## Results

### Principal component analysis

PCA yielded 10 principal components (PCs) that together accounted for 65% of the variation in the data set. The top 10 PCs were selected based on the scree plot, eigenvalues, and parallel analysis, as well as consideration of content and theoretical consistency. To interpret the dietary patterns represented by these PCs, we focused on the top and bottom 10th percentile of the PC loadings for each food category (Table 1). Both the magnitude and direction of the loadings were interpreted. For example, the first principal component (PC1) has high positive loadings of organ meats (e.g., liver and other organ meats), fish, chili peppers, and gravy, indicating that participants who followed the dietary pattern represented by PC1 typically consumed greater amounts of these foods. The second principal component (PC2) represents a healthy dietary pattern with high consumption of fruits and vegetables (green, dark-yellow, and other) and low consumption of liquor, soft drinks, beer, and gravy. In contrast, the third principal component (PC3) characterizes a relatively unhealthy dietary pattern with high consumption of high-fat dairy, red meat, refined grains, sweets and low consumption of meal replacements, organ meats, liquor, and wine. Additionally, PC4 identifies a large number of liquids with positive loadings of juice, fruit juices, and soft drinks as well as negative loadings of coffee and tea. PC4 also has large negative loadings of non-dairy cream and sweets. PC5 has high positive loadings of vegetables, salad dressing, and beer as well as large negative loadings of low-fat dairy, sweets, whole grains, and margarine. PC 6 represents a dietary pattern with high consumption of low-fat dairy and alcoholic beverages (e.g., liquor, beer, wine). The remaining principal components can be interpreted in a similar way based on the sign and absolute value of the PC loadings.

**Table 1:** Top and bottom 10th percentile of PC loadings for given food categories

Food Category	PC1	PC2	PC3	PC4	PC5	PC6	PC7	PC8	PC9	PC10
Low-fat Dairy	0.055	.	.	.	− 0.475	0.312	− 0.266	.	.	.
High-fat Dairy	.	.	0.318	.	.	.	0.282	.	.	.
Non-dairy Cream	.	.	.	−0.354	.	−0.227	.	− 0.266	.	.
Meal Replacement	.	.	−0.213	.	.	.	.	.	.	.
Red Meats	.	.	0.298	.	.	.	.	.	0.222	−0.286
Processed Meats	.	.	.	.	.	.	.	.	0.189	.
Organ Meats	0.239	.	−0.19	.	.	.	.	.	.	.
Poultry	.	.	.	.	.	.	−0.164	0.334	.	−0.367
Fish	0.211	.	.	.	.	.	.	0.206	.	−0.245
Eggs	.	.	.	.	.	.	.	.	0.233	.
Soups	.	.	.	.	.	.	.	.	.	.
Refined Grains	.	.	0.376	.	.	.	.	.	.	.
Sweets	0.059	.	0.306	−0.365	−0.271	.	.	.	−0.237	.
Snacks	.	.	.	.	.	.	.	0.188	.	0.293
Nuts	.	.	.	.	.	.	.	.	.	0.365
Whole Grains	.	.	.	.	−0.413	.	.	.	.	.
Fruit	.	0.335	.	.	.	.	.	.	.	.
Juice	.	.	.	0.163	.	.	0.25	−0.234	.	−0.273
Legumes	.	.	.	.	.	−0.21	.	.	.	.
Chili Peppers	0.237	.	.	.	.	.	.	.	.	.
Potatoes	.	.	.	.	.	.	.	.	.	0.176
Green, leafy vegetables	.	0.302	.	.	0.236	.	.	.	.	.
Dark-yellow Vegetables	.	0.248	.	.	.	.	.	.	.	.
Tomatoes	.	.	.	0.166	.	.	.	.	−0.283	.
Other Vegetables	.	0.34	.	.	0.23	.	.	.	.	.
Margarine	.	.	.	.	−0.254	− 0.211	− 0.394	− 0.227	.	0.308
Butter	.	.	.	.	.	.	0.499	.	0.358	.
Salad Dressings	.	.	.	.	0.228	.	.	.	.	.
Coffee	0.068	.	.	−0.592	.	.	.	− 0.318	.	.
Tea	.	.	.	−0.273	.	−0.372	.	0.404	−0.436	.
Liquor	.	−0.202	− 0.16	.	.	0.241	.	.	.	.
Fruit Juices	.	.	.	0.224	.	.	0.273	.	−0.273	.
Soft Drinks	0.047	−0.238	.	0.14	.	.	−0.283	.	.	.
Beer	.	−0.208	.	.	0.198	0.377	.	.	.	.
Wine	.	.	−0.221	.	.	0.276	.	.	.	.
Gravy	0.215	− 0.188	.	.	.	.	.	.	.	.

Note: food categories were based on those used by Kerver et al. [6]

### Linear regression with selected principal components

The 10 selected principal components were included in the multiple linear regression model to predict the levels of triglycerides, LDL cholesterol, HDL cholesterol, and total cholesterol. The regression model was first estimated using the training set and then evaluated using the test set. During the training stage, the regression model was statistically significant for triglycerides (*F*(18, 760) = 6.04, *p* < .001), LDL cholesterol (*F*(18, 736) = 3.85, *p* < .001), HDL cholesterol (*F*(18, 1617) = 6.04, *p* < .001), and total cholesterol (*F*(18, 1617) = 7.21, *p* < .001). To avoid model overfitting, model performance was evaluated using the independent test set. The correlation coefficient between the observed outcome and the predicted outcome for the test data was 0.43, 0.17, 0.50, and 0.21 for triglycerides, LDL cholesterol, HDL cholesterol, and total cholesterol respectively (Table 2). Additionally, adjusted *R*^2^, the modified version of *R*^2^ that has been adjusted for the number of predictors in the model, was calculated for each regression model using the test data (adjusted *R*^2^ = 0.163, 0.005, 0.235, and 0.024 for triglycerides, LDL cholesterol, HDL cholesterol, and total cholesterol respectively).

**Table 2:** Model comparison between linear regression with selected principal components and the LASSO model in terms of adjusted *R*^2^ and correlation coefficient *r*

	Triglycerides		LDL cholesterol		HDL cholesterol		Total cholesterol	
	Adjusted *R*^*2*^	r	Adjusted *R*^*2*^	r	Adjusted *R*^*2*^	r	Adjusted *R*^*2*^	r
Regression with PCs	0.163	0.43	0.005	0.17	0.235	0.50	0.024	0.21
LASSO	0.861	0.93	0.899	0.95	0.890	0.95	0.935	0.97

### The LASSO model

The LASSO model was directly applied to the defined food categories to predict the levels of triglycerides, LDL cholesterol, HDL cholesterol, and total cholesterol. The model tuning parameter lambda was automatically selected using cross-validation to zero out the coefficients of categories not helpful for predicting the outcomes. For triglycerides, the model included 12 predictors with non-zero coefficients and yielded a correlation coefficient of 0.93 and adjusted *R*^2^ of 0.861 for the independent test set (Table 2). For LDL, the model identified 18 predictors with non-zero coefficients and produced a correlation coefficient of 0.95 and adjusted *R*^2^ of 0.899 for the test set. For predicting HDL and total cholesterol, the model selected 25 and 29 predictors. As shown in Table 2, the correlation coefficients calculated using the test set were 0.95 and 0.97 for HDL and total cholesterol respectively. The corresponding adjusted *R*^2^ values were 0.890 and 0.935.

### Triglycerides

In the multiple regression with selected principal components, significant predictors for triglycerides were PC2 (*b* = − 0.037, *t* = − 2.99, *p* = 0.003), and PC3 (*b* = 0.039, *t* = 3.12, *p* = 0.002). This makes sense because PC2 represented a healthy dietary pattern with high consumption of fruits and vegetables and low consumption of liquor, soft drinks, beer, and gravy. Thus, the dietary pattern represented by PC2 was negatively associated with the level of triglycerides. PC3 characterized a relatively unhealthy dietary pattern with high consumption of high-fat dairy, red meat, refined grains, sweets and low consumption of meal replacements, organ meats, liquor and wine. Hence, the dietary pattern denoted by PC3 was positively associated with the level of triglycerides. Consistent with the findings from multiple regression analysis, the LASSO model suggested that increased consumption of meal replacements, organ meat, chili, dark-yellow vegetables, tea, and wine was negatively associated with the level of triglycerides.

### LDL cholesterol

Multiple regression analysis with selected principal components yielded significant predictors for LDL cholesterol, including PC4 (*b* = − 0.025, *t* = − 2.55, *p* = 0.011) and PC6 (*b* = − 0.032, *t* = − 2.76, *p* = 0.006). This suggests that both PC4 and PC6 negatively correlated with the level of LDL cholesterol. PC4 contained large negative loadings of non-dairy cream and sweets. PC6 represented a dietary pattern with high consumption of low-fat dairy and alcoholic beverages (e.g., liquor, beer, wine). Similar findings were suggested by the LASSO model. From Table 3, we can see that high consumption of low-fat dairy, meal replacements, organ meats, soups, chili, liquor, and wine predicted a decreased level of LDL cholesterol, while high consumption of high-fat dairy, red meat, refined grains, and sweets predicted an increased LDL cholesterol level.

**Table 3:** Estimated coefficients for the individual food categories according to the LASSO model

Food category	Triglycerides	LDL cholesterol	HDL cholesterol	Total cholesterol
(Intercept)	86.702	85.593	97.060	101.835
Lfat_dairy	.	−0.002	.	−0.001
Hfat_dairy	.	0.007	0.037	0.045
Non_dairy_cream	−0.006	.	−0.007	− 0.013
Meal_repl	−0.058	− 0.087	− 0.052	−0.066
Red_meat	.	0.001	.	0.043
Processed_meat	.	.	.	.
Organ_meats	−0.333	−0.383	−0.394	− 0.472
Poultry	.	.	.	.
Fish	.	.	.	.
Eggs	.	.	−0.011	−0.011
Soups	.	−0.017	−0.005	− 0.021
Refined_grains	.	0.010	0.019	0.031
Sweets	.	0.011	.	0.020
Snacks	.	.	.	−0.003
Nuts	.	.	−0.011	− 0.015
Whole_grains	.	.	−0.006	−0.005
Pizza	.	.	.	−0.001
Fruit	.	.	.	.
Juice	.	.	−0.006	−0.009
Legumes	.	.	−0.012	−0.025
Chili	−0.039	−0.105	− 0.128	−0.137
Potatoes	.	.	.	.
Green_leafy_veg	.	.	0.002	.
Cruciferous_veg	.	.	.	.
Darkyellow_veg	−0.021	.	.	.
Tomatoes	.	.	.	0.013
Other_veg	.	.	0.034	0.036
Margarine	.	−0.024	−0.018	−0.027
Butter	.	−0.019	−0.013	− 0.024
Salad_dressings	.	.	0.007	.
Coffee	.	.	.	.
Tea	−0.002	.	.	.
Liquor	.	−0.032	−0.011	− 0.013
Fruit_juices	.	.	.	.
Soft_drinks	.	.	−0.010	−0.001
Beer	.	.	.	.
Wine	−0.057	− 0.021	−0.006	− 0.038
Gravy	−0.011	− 0.029	−0.041	− 0.048
Age	0.011	0.010	0.009	0.011
Bmi	0.042	0.021	0.004	0.020
Income	0.068	0.063	0.040	0.053
Sex	.	.	.	.
Eth1	.	.	.	.
Eth2	.	.	.	.
Eth3	.	.	.	.
Eth4	.	.	.	.

*.* = Coefficient shrunk to zero

### HDL cholesterol

Significant predictors for HDL cholesterol in the multiple regression model were PC2 (*b* = 0.008, *t* = 2.11, *p* = 0.035), PC3 (*b* = − 0.009, *t* = − 2.29, *p* = 0.022), PC4 (*b* = − 0.015, *t* = − 2.86, *p* = 0.004), PC5 (*b* = 0.029, *t* = 5.38, *p* < .001), and PC6 (*b* = 0.040, *t* = 6.43, *p* < .001). This suggests that the level of HDL cholesterol, known as “good” cholesterol, positively correlated with high consumption of fruits and vegetables (represented by PC2) and low consumption of high-fat dairy, sweets and red meats (characteristics of PC3). Additionally, increased consumption of vegetables, salad dressing and beer (denoted by PC5) as well as low-fat dairy and alcoholic beverages (PC6) predicted an increased HDL cholesterol level. PC4 represented a dietary pattern with high consumption of a number of liquids (e.g., juice, fruit juices and soft drinks) and low consumption of non-dairy cream and sweets. Hence, the dietary pattern characterized by PC4 was negatively associated with the level of both “good” and “bad” cholesterol levels. Results from the LASSO model suggest that increased consumption of vegetables and decreased consumption of a number of liquids (e.g., juice and soft drinks) predicted increases in the level of HDL cholesterol. Additionally, consumption of proteins such as organ meats, nuts, chili peppers, and eggs predicted decreases in HDL, while high-fat dairy and refined grains predicted increases in HDL level.

### Total cholesterol

For total cholesterol levels, PC3 (*b* = 0.007, *t* = 2.41, *p* = 0.016), PC4 (*b* = − 0.013, *t* = − 3.07, *p* = 0.002), and PC5 (*b* = 0.016, *t* = 3.79, *p* < .001) were significant predictors in the multiple regression model. As described previously, PC3 represented a relatively unhealthy dietary pattern with high consumption of high-fat dairy, red meat, refined grains, and sweets. Thus, PC3 was positively associated with the level of total cholesterol. The dietary pattern characterized by high consumption of a number of liquids and low consumption of non-dairy cream and sweets (PC4) was negatively associated with the level of total cholesterol. Additionally, PC5, characterized by high consumption of vegetables, salad dressing and beer, was positively related to the level of total cholesterol. Results from the LASSO model suggested similar findings. The increased consumption of high-fat dairy, red meat, sweets, and certain vegetables (e.g., peppers, green beans) predicted an increase in total cholesterol, while higher consumption of non-dairy cream, meal replacements, organ meats, legumes, liquor and soft drinks predicted a decrease.

## Discussion

The study marks the first application of the LASSO model, a powerful analytic tool, to the identification of dietary patterns predictive of cardiovascular disease related risk factors. Results show that LASSO achieved higher prediction accuracy and explained a much larger percent of variability in all four risk factors than did a multiple regression model with derived principal components. The major limitation of PCA in dietary pattern identification is that patterns explaining most of the variation in diet intake don’t necessarily explain an equivalent amount of variation in the outcome variable. This is because PCA uses only the covariance matrix of predictors without taking the outcome variable into consideration. In general, PCA, factor and cluster analyses are not prediction techniques; therefore, identified patterns are not guaranteed to be predictive of specific health outcomes. In contrast, the LASSO model performs variable selection and prediction simultaneously, resulting in a sparse model including only food groups predictive of the outcome.

Despite their differences, both models demonstrate the ability to find predictive and meaningful dietary patterns. For example, the loadings matrix of the PCA demonstrates that participants may have distinct dietary patterns: there are high fruit and vegetable consumers (PC2), high comfort-food consumers (PC3), and high alcohol consumers (PC6), among others. Additionally, the identified dietary patterns showed differential relationships with the four biomarkers for cardiovascular disease. For instance, PC3, characterized by high consumption of high-fat dairy, red meat, refined grains and sweets, was positively associated with triglyceride level and total cholesterol and negatively associated with HDL cholesterol. A healthy dietary pattern represented by PC2, with high consumption of fruit and vegetables, negatively correlated with triglyceride level and positively correlated with HDL cholesterol level. In this way, PCA can be a useful and interpretable tool for extracting patterns from the data. However, interpretation of identified principal components is not always straightforward, as some food categories belonged to multiple principal components. For example, PC2 and PC5 both contained high positive loadings of green leafy vegetables and other vegetables. The LASSO model is typically more interpretable, since individual food categories rather than principal components are included as predictors and have their own estimated coefficients to indicate their effect on the outcome. Meal replacements and organ meats, for example, were consistently estimated to have negative coefficients across all four biomarkers.

Comparison of selected food groups by multiple regression and the LASSO model showed mixed results. For example, PC2 was negatively associated with triglycerides, indicating that increased consumption of fruit and vegetables predicted decreases in triglyceride level. The LASSO model for the same outcome only selected fruit and leafy vegetables as relevant variables, shrinking the coefficient for other vegetables (e.g., peppers, green beans) to zero. This level of sparsity may contribute to LASSO’s better prediction. A small handful of counterintuitive results were, however, found for the LASSO model, such as the prediction that increased intake of butter and margarine might lower total cholesterol levels. Note that the LASSO model included multiple types of foods in predicting the outcome; thus the estimated coefficient for an individual food category represented the effect of this category on the outcome variable assuming the consumption of other foods stayed the same.

To further explain these findings, consider that total cholesterol is itself composed of multiple types of chemicals, mostly LDL and HDL cholesterol, commonly known as “bad” and “good” cholesterol respectively. The effect of the food categories on total cholesterol level may be somewhat difficult to interpret due to the presence of both good and bad cholesterol. In essence, each component will be adding noise to the measurement of the other, and this noise may be detectable in a very sensitive system. Hence, the LASSO model included many more categories of foods for predicting total cholesterol than for LDL cholesterol and triglycerides.

There are a number of considerations for future investigation. The current literature suggests that analytical tools based on underlying patterns are not easily replicable [7, 12], and thus some of the algorithms discussed here are unlikely to do well in varied environments. Some investigations have rendered other “hybrid methods” that compromise between hypothesis-driven methods and data-driven methods similar to LASSO. One such method is the reduced rank regression [18], whose strength lies in its flexibility in accounting for as much variance in the outcome variable as possible. It is comparable to LASSO inasmuch as both involve automatic variable selection. One disadvantage of reduced rank regression, however, is that the number of selected components must be less than or equal to the number of outcome variables, which may limit its potential applications.

There also exist further iterations of the LASSO model such as the sparse group LASSO [25]. This approach, similar to LASSO as used here, performs automatic variable selection in the context of regression using a shrinkage parameter, but has the advantages of group assignment and group-level selection [25–27]. This method allows for the original individual predictors to be defined as groups of correlated predictors; ultimately, it performs variable selection at both the individual predictor and the group levels. Future research could apply sparse group LASSO to examine the issue of how to best define food groups based on Food Frequency Questionnaire raw scores.

## Conclusions

In summary, the LASSO model is shown to be an appropriate and promising statistical method to derive dietary patterns predictive of diseases or health outcomes. To the best of our knowledge, LASSO has not been applied to identify and examine dietary patterns predictive of health outcomes. Hence, our application and evaluation of the LASSO model in this context is innovative and novel. However, the usefulness of LASSO needs to be confirmed in future studies involving other diseases and risk factors such as some new emerging risk biomarkers in cardiovascular diseases and disorders (e.g., uric acid, growth-differentiation factor-15) [28]. We acknowledge the limitation of using levels of triglycerides, LDL, HDL and total cholesterol in the blood to predict cardiovascular disease risk. A more comprehensive list of risk factors for cardiovascular disease should be examined in future studies. Additional confounding covariates such as smoking status and social economic status should be considered.

## Abbreviations

BMI: Body mass index
FFQ: Food frequency questionnaire
HEI: Healthy eating index
LASSO: Least absolute shrinkage and selection operator
PCA: Principal component analysis
